# The Role of *Vascular Endothelial Growth Factor A* Polymorphisms in Breast Cancer

**DOI:** 10.3390/ijms131114845

**Published:** 2012-11-13

**Authors:** Doonyapat Sa-nguanraksa, Pornchai O-charoenrat

**Affiliations:** Division of Head-Neck and Breast Surgery, Department of Surgery, Faculty of Medicine, Siriraj Hospital, Mahidol University, Bangkok 10700, Thailand; E-Mail: doonyapat051@yahoo.com

**Keywords:** angiogenesis, breast cancer, polymorphism, vascular endothelial growth factor

## Abstract

Breast cancer is the most common cancer in females and the leading cause of cancer death in women worldwide. Angiogenesis, the formation of new blood vessels, plays an important role in the development and progression of breast cancer. Vascular endothelial growth factor A (VEGFA), the key modulator of angiogenesis, is highly expressed in cancer tissue and correlates with its more aggressive features. Polymorphisms of *VEGFA* alter the levels of expression and subsequently influence the susceptibility and aggressiveness of breast cancer. Assessment of *VEGFA* polymorphisms may be used for the identification of patients suitable for anti-VEGFA therapy.

## 1. Breast Cancer

Breast cancer is the most common cancer and the leading cause of cancer death in women worldwide, accounting for 23% of the total new cancer cases and 14% of the total cancer deaths in 2008 [1]. Several factors contribute to initiation and progression of breast cancer. Increased exposure to estrogen is correlated with increased risk for breast cancer. Changes in physical activity, reproductive patterns, obesity and increased pollution in Asian countries has led to increased incidences of breast cancer. The hallmarks of cancer comprise sustaining proliferative signaling, evading growth suppressors, resisting cell death, enabling replicative immortality, inducing angiogenesis, and activating invasion and metastases [2]. Genetic deregulation leads to progression of these distinctive capacities. Accumulation of genetic mutation results in increased aggressive phenotypes. High penetrance genes, such as *BRCA1*, *BRCA2* and *p53* are associated with breast cancer risk with relative risk (RR) higher than 10. However, variations in these genes account for 0.1% in the population and have impact only on hereditary breast cancer. The vast majority of breast cancer cases are sporadic and not related to high-penetrance genes. Combination of polymorphisms in low-penetrance genes may play an important role in breast cancer initiation and progression.

## 2. Angiogenesis in Breast Cancer

Cancer cells require nutrients, oxygen and the ability to evacuate waste products. These requirements are accomplished by means of angiogenesis, the formation of new vascular networks from pre-existing blood vessels. This process is one of the crucial factors that contributes to breast cancer growth and metastasis. Lack of tight controls in cancer angiogenesis results in a difference between normal and cancer angiogenesis [3]. The intra-tumoral vessels are irregular, tortuous, tapering, saccular and have numerous arteriovenous anastomosis or blind ends. The irregular architecture results in inadequate perfusion, leading to selection of more aggressive cancer cells and ineffective delivery of anticancer agents. Fenestrated endothelial lining without basement membrane leads to higher permeability. This site serves as cancer cell intravasation and subsequently promotes metastasis [4–6]. Analysis of tumor vasculature in *neuT* transgenic mice revealed that both sprouting and intussusception were involved in breast cancer angiogenesis [7]. Hyperplastic murine breast papillomas and histologically normal lobules adjacent to cancerous breast tissue can induce angiogenesis [8, 9]. These evidences suggest that angiogenesis precedes transformation of mammary hyperplasia to malignancy. In human, high microvessel densities (MVD) in premalignant lesions have been associated with high risk of future breast cancer [10]. High MVD in invasive disease has been correlated with a greater likelihood of metastatic disease and a shorter relapse-free survival (RFS) and overall survival (OS) in patients with node-negative breast cancer [11, 12]. Meta-analysis of 43 independent studies (8,936 patients) showed that high MVD predicted poor survival with relative risk (RR) = 1.54, 95% CI =1.29–1.84 for both DFS and OS. High MVD also significantly predicted poor survival for node-negative patients with RR 1.99, 95% CI =1.33–2.98 for DFS (2,727 patients) and RR = 1.54, 95% CI =1.01–2.33 for OS (1,926 patients) [13]. Several molecules including fibroblast growth factor, platelet-derived growth factor, transforming growth factor-β participate in this complex process and one of the most important key modulators is vascular endothelial growth factor (VEGFA).

## 3. VEGFA Biology

**Figure 1:**
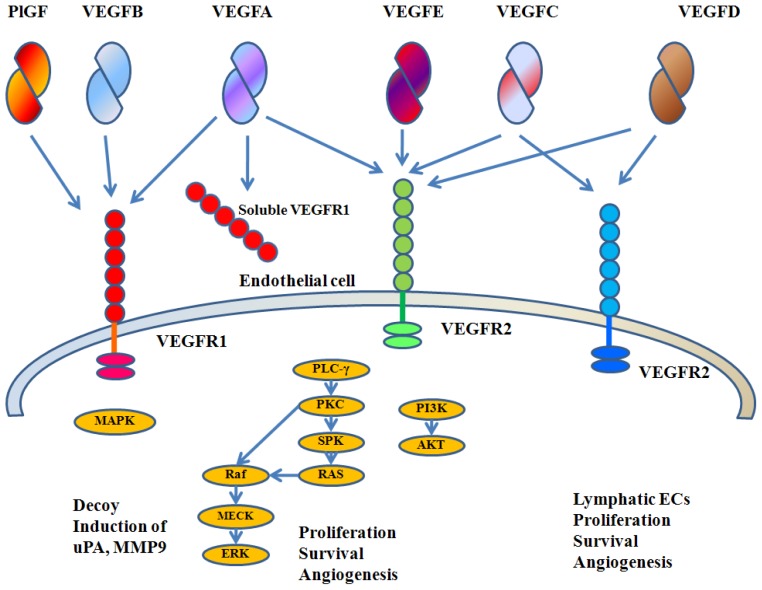
VEGF family and receptors. VEGFA binds both VEGFR-1 and VEGFR-2. PlGF and VEGFB bind only VEGFR-1. VEGFC and VEGFD bind VEGFR-2 and VEGFR-3. VEGFR-2 is the major mediator of EC mitogenesis and survival. VEGFR-1 does not mediate an effective mitogenic signal in EC and it may perform an inhibitory role by sequestering VEGFA and preventing its interaction with VEGFR-2.

VEGFA is a member of the VEGF family, which comprises VEGFA, VEGFB, VEGFC, VEGFD, and placental growth factor (PlGF). The VEGF family and receptors are summarized in Figure 1. The human *VEGFA* is localized in chromosome 6p21.3 and organized as eight exons separated by seven introns [14–16]. Alternative exon splicing was initially shown to result in the generation of four different isoforms (VEGFA_121_, VEGFA_165_, VEGFA_189_ and VEGFA_206_), having respectively 121, 165, 189 and 206 amino acids, after signal sequence cleavage [15, 16]. VEGFA_165_, the predominant isoform, lacks the residues encoded by exon 6, whereas VEGFA_121_ lacks the residues encoded by exons 6 and 7. Less frequent splice variants also have been reported, such as VEGFA_145_ and VEGFA_183_[17].

A well-documented *in vitro* activity of VEGFA is the ability to promote growth of endothelial cells (ECs) derived from arteries, veins and lymphatic vessels. VEGFA induces a potent angiogenic response in a variety of *in vivo* models [18, 19]. VEGFA delivery also induces lymphangiogenesis in mice [20]. Although ECs are the primary target of VEGFA, several studies have reported mitogenic effects on certain non-EC types [21]. VEGFA is a survival factor for ECs, both *in vitro* and *in vivo. In vitro*, VEGFA prevents apoptosis induced by serum starvation. Gerber *et al.* showed that such activity is mediated by the PI3-kinase/Akt pathway [22]. VEGFA also induces expression of the anti-apoptotic proteins Bcl-2 and A1 in ECs [23]. *In vivo*, the prosurvival effects of VEGFA are developmentally regulated. VEGFA inhibition results in extensive apoptotic changes in the vasculature of neonatal but not in that of adult mice [24]. Furthermore, a marked VEGFA dependence has been shown in ECs of newly formed but not of established vessels within tumors [25, 26]. Coverage by pericytes has been proposed to be one of the key events resulting in loss of VEGFA dependence [25].

VEGFA is also known as vascular permeability factor, based on its ability to induce vascular leakage [27, 28]. It is now well established that such permeability-enhancing activity underlies significant roles of this molecule in inflammation and other pathological circumstances. VEGFA induces an increase in hydraulic conductivity of isolated microvessels; this effect is mediated by increased calcium influx [29]. Consistent with a role in the regulation of vascular permeability, VEGFA induces endothelial fenestration in some vascular beds [30]. In addition, VEGFA induces vasodilatation *in vitro* in a dose-dependent fashion as a result of EC-derived nitric oxide [31], and produces transient tachycardia, hypotension and a decrease in cardiac output when injected intravenously in conscious, instrumented rats [32].

In 1993, Kim *et al.* reported that antibodies to VEGFA exerted a potent inhibitory effect on the growth of several cancer cell lines in nude mice [33]. Subsequently, many other cancer cell lines were found to be inhibited *in vivo* by these antibodies and other anti-VEGFA treatments, including small-molecule inhibitors of VEGFR signaling, antisense oligonucleotides and antibodies to VEGFR-2 [34]. Although cancer cells are the major source of VEGFA, cancer-associated stroma is also an important site of VEGFA production [35–37]. Proteolytic cleavage mediated by matrix metalloproteinase-9 results in enhancement of the activity of low, constitutive VEGFA, by making it available to bind VEGFR-2 [38].

## 4. Roles of VEGFA in Breast Cancer

Several lines of evidence implicate the importance of VEGFA in breast cancer [39]. Patients with locoregional ductal cancers have elevated serum VEGFA concentrations in comparison with women with benign breast tumors. The highest concentrations of serum VEGFA were founded in metastatic breast cancer, in particular among patients who did not receive cancer therapy for metastatic disease [40]. A significant correlation between VEGFA concentration and MVD has been reported [41]. A study in 29 invasive breast carcinomas revealed that VEGFA expression in the peritumoral ECs correlated with angiogenesis, lymphangiogenesis and higher pathologic stage. A study in 574 node-negative breast cancer patients showed that high VEGFA levels in tumor tissue were associated with larger tumor size, older age, and negative progesterone receptor (PR). Patients with low VEGFA had higher DFS and OS (84% *vs.* 75% and 93% *vs.* 86%, respectively). Tissue microarray of 642 breast cancers demonstrated that high levels of VEGFA and its receptors- VEGFR-1, VEGFR-2, and NRP-1 were significantly associated with poor survival. This study also demonstrated the correlation between the expression of VEGFA and its receptors within tumor cells, supporting an autocrine and paracrine function of VEGFA [42]. In a study of 50 invasive ductal carcinomas, expressions of VEGFA protein and mRNA were correlated with tumor size, lymph node metastasis and TNM staging. The MVD counts were correlated with the expression of VEGFA and axillary lymph node metastasis [43]. Cox analysis revealed that intratumoral VEGFA was an independent prognostic factor for node-negative breast cancer [44]. High VEGFA and low soluble VEGFR-1 levels (an intrinsic negative counterpart of VEGFA) were significantly associated with poor prognosis [45, 46]. Randomized study of tamoxifen adjuvant treatment in postmenopausal patients revealed that the patients with positive VEGFA had no response to tamoxifen [47]. In advanced breast cancer patients receiving bevacizumab and vinorelbine who had lower baseline plasma VEGFA before treatment had longer time to progress (9.3 months *vs.* 3.7 months) than those with higher plasma VEGFA [48]. This evidence supports the crucial role of VEGFA in the transition from benign to malignant breast disease and breast cancer aggressiveness.

## 5. *VEGFA* Polymorphisms

Genetic polymorphisms are common DNA sequence variations in the general population. This type of variation is different from mutation as the prevalence is higher than one percent and does not cause overt disease. Polymorphisms in the promoter region or exons may affect gene expressions or protein functions and influence different characteristics among individuals, such as height and skin color. Single nucleotide polymorphism (SNP), a single base difference among individual, is the most common type of genetic variation in the human genome. SNPs occur about once every 1000 base pairs. Association studies of genetic polymorphisms implicate the role of polymorphisms in susceptibility and aggressiveness of cancer.

**Figure 2:**
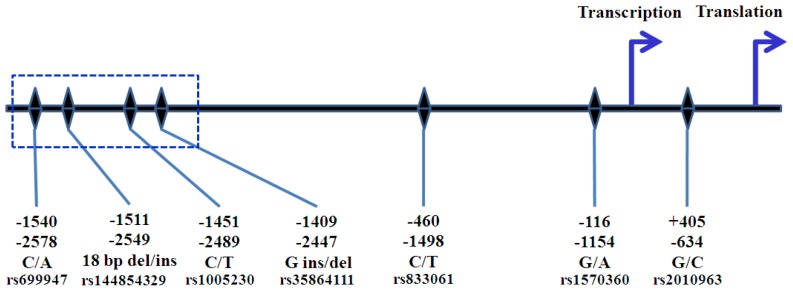
Nomenclature of *VEGFA* SNPs. The positions in the upper row correspond to the transcription start site, while the positions in the lower row correspond to the translation start site. The polymorphisms in the box are in complete LD. −2578C is linked with −2549 18 base pairs deletion, −2489C. and −2447G insertion. −2578A is linked with −2549 18 base pairs insertion, −2489T and −2447G deletion.

Several SNPs in the promoter and 5′ UTR of *VEGFA* have been identified [49, 50]. Nomenclature of these SNPs corresponds to either transcription or translation start site (Figure 2). The common sites of SNPs are at position −2578, −1498 −1154, and −634. Transitions of C/A at nucleotide position −2578 relative to the translation start site (−2578 C/A), −1455 T/C, −1154 G/A, −1001 G/C, and −7 C/T were reported. Individuals with the A allele at position −2578 also had an insertion of 18 nucleotides at position −2549, whereas CC homozygotes did not contain this insertion [49]. The −2578A was in complete linkage disequilibrium (LD) with −2447 del G and -2489T, while −2578C was linked with −2447G and −2489C [51]. Awata *et al.* identified seven SNPs in the promoter region and 5′ and 3′ UTR of *VEGFA* in Japanese population. Among these SNPs, the −634 G/C differed significantly between type II diabetes patients without retinopathy and those with any retinopathy, and the -634C allele was significantly associated with the presence of retinopathy. In addition, serum VEGFA levels were significantly higher in healthy subjects with the −634 CC genotype [52]. However, *in vitro* experiment using lipopolysaccharide (LPS)-stimulated peripheral blood mononuclear cell (PBMC) demonstrated that the −634 GG correlated with higher VEGFA production [50]. A study by Shahbazi *et al.* indicated that the −1154G and −2578C allele were associated with higher VEGFA production by PBMC and acute renal allograft rejection [53]. In non-small cell lung cancer, a low VEGFA expression in cancer tissues was significantly associated with the presence of the −2578 CC, −634 GG and −1154 AA and GA genotypes [54]. *In vitro* model suggested a haplotypic effect of the polymorphic *VEGFA* promoter on both basal and stimulated promoter activity [55]. However, this approach cannot exclude the interaction between polymorphisms in different positions. Results from our study (unpublished data), by utilizing site-directed mutagenesis approach, showed that alteration of −634G to C resulting in increased basal promoter activity. However, no transcription factor binding motif was identified at this position [56]. Identification of transcription factor binding site using TFSEARCH (version 1.3; Parallel Application TRC Laboratory, Real World Computing Partnership (RWCP): Tsukuba, Japan), revealed that −634G was the potential binding site for myeloid zinc finger protein 1 (MZF1) [50], which was expressed in hematopoietic progenitor cells that are committed to myeloid lineage differentiation [57]. Watson *et al.* reported that alteration from G to C diminished the potential binding capacity [50]. MZF1 might not have any role in breast cancer cells and transcription factor binding motif predicted by MatInspector Online Tool (Genomatix Software GmbH: Munich, Germany) found no potential transcription factor binds this position [52]. Transcriptional activity assessed in human glioma cell line (GI-1) and human lymphoblastic T-lymphocyte cell line (Jurkat) revealed that construction bearing −1154G/−634C haplotype had higher luciferase activity than −1154G/−634G haplotype [58]. This agreement may indicate direct effect of alteration from G to C at −634 position on promoter activity. Polymorphisms at this position may regulate promoter activity via posttranscriptional level. G to C alteration may affect IRES and enhance transcription of large VEGFA isoform [59]. Alteration of C to T at position 936 in 3′ UTR had protective effect against breast cancer and had a trend of correlation with lower plasma VEGFA levels [60]. Alteration from C to T at this position might lead to loss of potential AP-4 binding site or be in LD with other unknown downstream polymorphisms [61]. Analysis of mRNA stability in PBMC showed that transcript with 936C genotype was more stable than 936T genotype [62].

Polymorphisms on positions other than promoter and 5′ UTR were also reported. Abe *et al.* studied the correlation between 702 C/T, 936 C/T, and 1612 G/A SNPs on 3′ UTR and renal cell carcinoma in Japanese population but did not find any association [63]. Garcia-Closas *et al.* reported the protective effect of polymorphisms in intron 2, 1378 C/T. CT genotype had protective effect against bladder cancer in Spanish population when compared to CC genotype (OR = 0.65, 95% CI 0.46–0.91, *p* = 0.012) [64]. However, there was no published study on its functional significant.

## 6. The Role of *VEGFA* Polymorphisms in Breast Cancer Risk and Aggressiveness

Several reports examined the role of *VEGFA* polymorphisms as summarized in Tables 1 and 2. The most common polymorphisms that had been studied were located in promoter, 5′ UTR, and 3′UTR: −2578 A/C, −1498 C/T, −1154 G/A, −634 G/C and 936 C/T. Study in American population by Jacobs *et al.* showed that −2578C allele was associated with increased risk for invasive breast cancer but not for *in situ* or overall breast cancer [65]. In contrast, Schneider *et al.* reported that −2578 AA genotype was associated with higher risk of breast cancer in Caucasian and African-American population [66]. A study in Polish, German and Swedish breast cancer patients showed that *VEGFA* −2578 AA was associated with a low grade tumor [51]. In −2578 CC patients with positive ER and PR had higher incidence of recurrent [67]. This was concordant with the functional role of −2578C allele which was associated with increased VEGFA production by PBMC [53].

Schneider *et al.* reported the association between −1498 CC and the increased risk of breast cancer [66], however, there was no study that supports the role of −1498 C/T polymorphisms in breast cancer susceptibility. Survival analysis in Chinese population showed that −1498 CC genotype tended to be associated with decreased OS [81]. Determination of VEGFA levels in breast cancer tissue, or serum did not show any association with −1498 C/T polymorphisms [69, 68]. These results indicated that the association reported by Schneider *et al.* might be a false positive due to LD between −1498C/T and other positions.

A study in Caucasian and African–American population by Oliveira *et al.* revealed that −634 CC genotype was associated with increased risk for breast cancer. Nevertheless, other studies of −634 G/C polymorphisms did not demonstrate any association with breast cancer susceptibility. For breast cancer aggressiveness, −634 CC genotype was associated with larger tumor size and high grade tumor in Swedish population [51]. This was concordant with a study by Balasubramanian *et al.* that reported the association between −634C allele and maximum size of invasive component in Caucasian population [69]. In contrast, Langsenlehner *et al.* reported the association between −634C allele with small tumor size in Austrian population [68]. Lu *et al.* reported that −634G allele was associated with decreased overall survival in Chinese population [81]. Our hospital based case-control study including 483 Thai breast cancer patients and 524 controls demonstrated significant association between −634 GC and CC genotype with breast cancer susceptibility and also aggressiveness. Determination of *VEGFA* mRNA levels in breast cancer tissue by Semi-quantitative reverse transcription PCR revealed that −634 CC genotype was significantly associated with highest *VEGFA* mRNA when compared to GG and GC genotype.

T allele at 936 position in 3′UTR was reported to be associated with reduced risk of breast cancer in Austrians, Turkish, Chinese and also Polish with *BRCA1* mutation [60, 70,74–76]. Recent large population base case-control study in a Chinese population and large study in a Polish population without *BRCA1* mutation could not demonstrate the association between 936C/T polymorphisms and breast cancer risk [77, 79]. Studies by Oliveira *et al.* and Etienne-Grimaldi *et al.* showed that the T allele at this position was associated with lower aggressive breast cancer [72, 80]. Krippl *et al.* demonstrated the association between the 936T allele and lower plasma VEGFA levels, but it was not statistically significant [60]. The other studies could not demonstrate the association (Table 3).

The polymorphisms in promoter and 5′ UTR are in highly LD. The association between particular polymorphisms and breast cancer susceptibility and aggressiveness might be due to linkage with other functional polymorphisms. Haplotype analysis revealed that having 2 copy number of −2578A/−634G haplotype was associated with lower tumor grade [51]. Jacobs *et al.* reported that −2578A/−1154A/−634G was associated with reduced risk of breast cancer in American population [65]. Balasubramanian *et al.* reported the association between −1498T/−634C/−7C/936C haplotype and reduced breast cancer risk in Caucasian population [69]. In a large case-control study in Chinese population (1184 controls and 1093 patients), −1498T/−634G/936T haplotype was associated with a reduced risk of breast cancer in premenopausal women [70]. However, the authors emphasized the role of 936 C/T polymorphisms, located on 3′UTR, more than the promoter region and 5′UTR. These two studies included the downstream polymorphisms (936 C/T), which were not in LD with the polymorphisms upstream to the coding region. The alleles that were included in haplotype analysis might be linked with other functional polymorphisms that were not assessed. Comparison of haplotype analysis is complicate due to difference in alleles including in haplotypes and software that was used to generate haplotype and determine haplotype frequency.

Meta-analysis of five *VEGFA* polymorphisms (−2578 A/C: 4,016 cases/4159 controls, −1498 C/T: 1616 cases/1721 controls, −1154 G/A: 3233 cases/3491 controls, −634 G/C: 5060 cases/5306 controls and 936 C/T: 8013 cases/8203 controls) by Wang *et al.* showed that all five polymorphisms were not associated with risk of breast cancer [84]. Meta-analysis of 936 C/T polymorphisms by Yang *et al.* (4973 case/5035 controls) including one study with *BRCA1* mutation patients did not support the role of 936 C/T polymorphisms in breast cancer [85]. Meta-analysis by Gu and Wang (5729 cases/5868 controls) including similar studies except one study that recruited *BRCA1* mutation patients was excluded. A large study in Polish population and a small study in Turkish population were included in this analysis and also did not find the association [86]. Meta-analysis of −634 G/C polymorphisms in solid cancer revealed that there was no association between these polymorphisms and risk of malignancy. Subgroup analysis showed no association with breast cancer [87].

The correlation between *VEGFA* polymorphisms and breast cancer susceptibility and aggressiveness remains inconsistence. Non-replication of genetic association results is common in several studies and may be due to difference in population, sample size, demography, and study design. Three meta-analyses, which recruited similar primary studies, suggested null results. However, common limitation of meta-analysis such as quality of primary study, lack of individual data for further analysis and small size of each subgroup may lead to insufficient power. In addition, the majority of primary studies are in Caucasians. Further well designed studies in different ethnicities are required for conclusion.

## 7. Potential Clinical Application of *VEGFA* Polymorphisms

Incorporation of bevacizumab, a humanized monoclonal antibody to VEGFA, in chemotherapy, is one of the rapidly evolving areas in the treatment of breast cancer. Without the assessment of VEGFA status, several studies showed significant benefit of anti VEGFA treatment. A study of 722 women with metastatic or locally recurrent breast cancer received paclitaxel or paclitaxel with bevacizumab revealed that addition of bevacizumab to paclitaxel increased median progression free survival (PFS) from 5.9 to 11.8 months. However, the median overall survival was similar (26.7 and 25.2 months) [88]. 736 breast cancer patients with locally recurrent or metastasis breast cancer were randomly assigned to docetaxel 100 mg/m^2^ plus either placebo or bevacizumab 7.5 or 15 mg/kg every three weeks. Combination of bevacizumab 15 mg/kg with docetaxel showed superior median PFS to placebo plus docetaxel (10.1 *vs.* 8.2 months, respectively). The response rates also increased with bevacizumab [89]. 585 triple negative metastatic breast cancer patients in the ATHENA study were analyzed for the efficacy of bevacizumab. Median time to progression was 7.2 months. Median overall survival was 18.3 months. Overall response rate was 49% with complete response in 10% [90]. On the other hand, a randomized phase III trial compared the efficacy and safety of capecitabine with or without bevacizumab in 462 patients did not demonstrate an improvement in progression-free survival [91]. The authors raised the issue of patient selection to identify the one who should benefit from anti-VEGFA therapy. Baseline plasma VEGFA measured in advanced breast cancer patients before treatment with bevacizumab and vinorelbine showed that patients who had lower baseline plasma VEGFA had longer time to progress (9.3 months in patients with VEGFA ≤32.6 pg/mL *vs.* 3.7 months in patients with VEGFA >32.6 pg/mL) [48]. This evidence suggested the importance of VEGFA assessment. However, several cell types can produce VEGFA and the levels may alter during inflammatory process. Determination of VEGFA expression in breast cancer tissue by immunohistochemistry can distinguish the source of VEGFA, but revealed null results [92– 94]. Association study of *VEGFA* polymorphisms in 180 advanced breast cancer patients treated with paclitaxel alone or paclitaxel plus bevacizumab and 183 untreated patients revealed that *VEGFA*-2578 AA genotype was associated with better median OS in the combination arm when compared with AC combined with CC genotype. The *VEGFA*-1154 AA genotype also demonstrated a better median OS when compared to GG combined with GA genotype in the combination arm [95]. In contrast, analysis of 127 metastatic breast cancer patients treated with bevacizumab combined or not with taxane-based chemotherapy did not support these findings but suggested the role of 936C/T polymorphisms as a marker of time to progression. The patients with 936 CT and 936 TT genotypes had a longer time to progression when compared with the one with the 936 CC genotype (11.5 *vs.* 9.7 months) [80]. The disagreement between these two studies might be due to different in primary end point and the former study did not investigate the impact of 936 C/T on breast cancer progression.

These findings indicated that methods for selection of the patients that are suitable for anti-VEGFA therapy should be developed and established. Assessment of *VEGFA* polymorphisms may be used for evaluation of breast cancer susceptibility, aggressiveness and identification of patients that are suitable for anti-VEGFA therapy. Further clinical trials, which include *VEGFA* polymorphism study including a large number of patients and different ethnicities, should be conducted.

## Figures and Tables

**Table 1:** Studies of *VEGFA* polymorphisms and susceptibility of breast cancer.

SNPs	Authors	Population	Case/control	Susceptibility
−2578C/A rs699947	Langsenlehner *et al.*[68]	Austrian	804/804	N.S.
	Jacobs *et al.*[65]	American	504/501	C allele was associated with increased risk of invasive cancer, OR = 1.46 (1.00–2.14), *p* (trend) = 0.049.
	Jin *et al.*[51]	Polish German Swedish	1525/1503	N.S.
	Schneider *et al.*[66]	Caucasian, African- American	175/520	AA genotype was associated with higher risk of breast cancer, OR = 1.99 (1.06–3.74), *p* = 0.03 (adjusted by Gail score).

−2489C/T rs1005230	Langsenlehner *et al.*[68]	Austrian	804/804	N.S.

−1498C/T rs833061	Balasubramanian *et al.*[69]	Caucasian	500/498	N.S.
	Langsenlehner *et al.*[68]	Austrian	804/804	N.S.
	Kataoka *et al.*[70]	Chinese	1184/1093	N.S.
	Schneider *et al.*[66]	Caucasian, African- American	175/520	CC genotype was associated with higher risk of breast cancer, OR = 2.01 (1.08–3.76), *p* = 0.03.

−1154G/A rs1570360	Jacobs *et al.*[65]	American	504/501	G allele was associated with increased risk of invasive cancer, OR = 1.64 (1.02–2.64), *p* = 0.007.
	Jin *et al.*[51]	Polish German	586/570	N.S.
	Schneider *et al.*[66]	Caucasian, African-American	175/520	N.S.
	Smith *et al.*[71]	English	263/144	N.S.

−634G/C rs2010963	Balasubramanian *et al.*[69]	Caucasian	500/498	N.S.
	Langsenlehner *et al.*[68]	Austrian	804/804	N.S.
	Jacobs *et al.*[65]	American	504/501	N.S.
	Jin *et al.*[51]	Swedish	941/936	N.S.
	Kataoka *et al.*[70]	Chinese	1184/1093	N.S.
	Oliveira *et al.*[72]	Caucasian, African-American	235/235	CC genotype was associated with increased risk for breast cancer, OR = 2.20 (1.20–4.02), *p* = 0.01 when compared to GG and GC genotype.
	Schneider *et al.*[66]	Caucasian, African-American	175/520	N.S.

−7C/T rs25648	Balasubramanian *et al.*[69]	Caucasian	500/498	N.S.
	Langsenlehner *et al.*[68]	Austrian	804/804	N.S.

rs833070 C/T (intron 2)	Beeghly-Fadiel *et al.*[73]	Chinese	4,419/1,851	TT genotype was associated with increased risk for breast cancer when compared to CC and CT genotype, OR = 1.26 (1.05–1.52), *p* = 0.016.

*SNPs*	*Authors*	*Population*	*Case/control*	*Susceptibility*

936C/T rs3025039	Balasubramanian *et al.*[69]	Caucasian	500/498	N.S.
	Krippl *et al.*[60]	Austrian	500/500	T allele had protective effect OR = 0.51, (0.38–0.70), *p* < 0.001.
	Langsenlehner *et al.*[68]	Austrian	804/804	N.S.
	Eroglu *et al.*[74]	Turkish	60/60	CT was more frequent in patient group (*p* = 0.001)
	Gerger *et al.*[75]	Austrian	500/500	T allele was associated with decreased risk of breast cancer, OR = 0.58 (0.44–0.76), *p* < 0.001.
	Jacobs *et al.*[65]	American	504/501	CC genotype was associated with reduce risk for *in situ* cancer, OR = 0.59 (0.37–0.93), *p* = 0.052.
	Jakubowska *et al.*[76]	Polish BRCA1 mutation carriers	190/319	CT and TT genotypes were associated with reduced risk of breast cancer, OR = 0.63 (0.41–0.98), *p* = 0.042.
	Jakubowska *et al.*[77]	Polish	1015/1073	N.S.
	Jin *et al.*[51]	Polish German Swedish	1519/1489	N.S.
	Kataoka *et al.*[70]	Chinese	1184/1093	TT genotype was associated with decreased risk of breast cancer in premenopausal women, OR = 0.45 (0.25–0.79), *p* = 0.041.
	Oliveira *et al.*[72]	Caucasian, African- American	235/235	N.S.
	Schneider *et al.*[66]	Caucasian, African- American	175/520	N.S.
	Rodrigues *et al.*[78]	Spanish	453/461	CT and TT genotypes had protective effect against breast cancer, OR = 0.67 (0.48–0.92), *p* (trend) = 0.014
	Zhang *et al.*[79]	Chinese	1918/1819	N.S.

1612G/A rs10434	Langsenlehner *et al.*[68]	Austrian	804/804	N.S.

N.S.: not significant.

**Table 2:** Studies of *VEGFA* polymorphisms and aggressiveness of breast cancer.

SNPs	Authors	Population	Case/control	Aggressiveness
−2578C/A rs699947	Jin *et al.*[51]	Polish, German, Swedish	1525/1503	AA genotype was associated with low grade tumor, *p* (trend) = 0.04.
	Langsenlehner *et al.*[68]	Austrian	804/804	N.S.
	Kidd *et al.*[67]	Caucasian	441/−	ER and PR positive patients with CC genotype had higher incidence of recurrent, *p* (trend) = 0.026
	Etienne-Grimaldi *et al.*[80]	Caucasian	137/−	N.S.

−2489C/T rs1005230	Langsenlehner *et al.*[68]	Austrian	804/804	N.S.

−1498C/T rs833061	Lu *et al.*[81]	Chinese	1193/−	CC genotype tended to be associated with decreased OS, HR = 1.5 (0.9–2.5), *p* (trend) = 0.11.
	Balasubramanian *et al.*[69]	Caucasian	500/498	N.S.
	Langsenlehner *et al.*[68]	Austrian	804/804	N.S.
	Etienne-Grimaldi *et al.*[80]	Caucasian	137/−	N.S.

−1154G/A rs1570360	Jin *et al.*[51]	Polish, German	586/570	N.S.
	Smith *et al.*[71]	English	263/144	AG was associated with good prognosis *, OR = 2.63 (1.12–6.20), *p* = 0.02. GG was associated with negative ER, OR = 0.35 (0.14–0.84), *p* = 0.02.
	Kidd *et al.*[67]	Caucasian	441/−	N.S.
	Etienne-Grimaldi *et al.*[80]	Caucasian	137/−	N.S.

*SNPs*	*Authors*	*Population*	*Case/control*	*Aggressiveness*

−634G/C rs2010963	Jin *et al.*[51]	Swedish	941/936	CC genotype was associated with tumor size > 20 mm, OR = 2.20 (1.27–3.82), *p* = 0.004, and higher histologic grade, *p* = 0.009.
	Lu *et al.*[81]	Chinese	1193/−	G allele was associated with decreased OS. HR = 1.6 (1.0–2.5) for GG genotype.
	Balasubramanian *et al.*[69]	Caucasian	500/498	C allele was associated with maximum size of invasive component, *p* (trend) = 0.02.
	Langsenlehner *et al.*[68]	Austrian	804/804	C allele was associated with small tumor size, *p* < 0.001.
	Oliveira *et al.*[72]	Caucasian/Afric an-American	235/235	N.S.
	Kidd *et al.*[67]	Caucasian	441/−	N.S.
	Etienne-Grimaldi *et al.*[80]	Caucasian	137/−	N.S.
	Beeghly-Fadiel *et al.*[73]	Chinese	Stage 1:1193/− Stage 2:5381/−	N.S.

−7C/T rs25648	Balasubramanian *et al.*[69]	Caucasian	500/498	N.S.
	Langsenlehner *et al.*[68]	Austrian	804/804	N.S.

936C/T rs3025039	Eroglu *et al.*[74]	Turkish	60/60	N.S.
	Lu *et al.*[81]	Chinese	1193/−	N.S.
	Wolf *et al.*[82]	Caucasian	37/−	Number of T allele was correlated with FDG uptake score, *p* (Spearman correlation) = 0.032.
	Balasubramanian *et al.*[69]	Caucasian	500/498	N.S.
	Krippl *et al.*[60]	Austrian	500/500	N.S.
	Langsenlehner *et al.*[68]	Austrian	804/804	N.S.
	Oliveira *et al.*[72]	Caucasian/African-American	235/235	CC genotype was correlated with increased risk for aggressive breast cancer, OR = 1.76 (1.10–2.90) and negative ER, OR = 0.86 (1.10–3.10).
	Knechtel *et al.*[83]	Caucasian	432/−	N.S.
	Etienne-Grimaldi *et al.*[80]	Caucasian	137/−	TT and CT genotypes were associated with longer time to progression when compared to 936 CC genotype (11.5 *vs.* 9.7 months, *p* = 0.022).

1612G/A rs10434	Langsenlehner *et al.*[68]	Austrian	804/804	N.S.

* According to Nottingham Prognostic Index; N.S.: not significant.

**Table 3:** Studies of *VEGFA* polymorphisms and levels of expression.

SNPs	Authors	Population	Case/control	Levels of expression	

				Specimen	Results
−2578C/A rs699947	Langsenlehner *et al.*[68]	Austrian	−/81	Plasma	N.S.

−2489C/T rs1005230	Langsenlehner *et al.*[68]	Austrian	−/81	Plasma	N.S.

−1498C/T rs833061	Balasubramanian *et al.*[69]	Caucasian	500/498	Serum/Tissue	N.S.
	Langsenlehner *et al.*[68]	Austrian	−/81	Plasma	N.S.

−634G/C rs2010963	Balasubramanian *et al.*[69]	Caucasian	500/498	Serum/Tissue	N.S.
	Langsenlehner *et al.*[68]	Austrian	−/81	Plasma	N.S.
	Oliveira *et al.*[72]	Caucasian, African-American	235/235	Serum	N.S.

−7C/T rs25648	Balasubramanian *et al.*[69]	Caucasian	500/498	Serum/Tissue	N.S.
	Langsenlehner *et al.*[68]	Austrian	−/81	Plasma	N.S.

936C/T rs3025039	Balasubramanian *et al.*[69]	Caucasian	500/498	Serum/Tissue	N.S.
	Krippl *et al.*[60]	Austrian	−/21	Plasma	Carriers of T allele tend to had lower VEGFA levels (not reach significant)
	Langsenlehner *et al.*[68]	Austrian	−/81	Plasma	N.S.
	Oliveira *et al.*[72]	Caucasian, African-American	235/235	Serum	N.S.

1612G/A rs10434	Langsenlehner *et al.*[68]	Austrian	−/81	Plasma	N.S.

N.S.: not significant.

## References

[1] Jemal A., Bray F., Center M.M., Ferlay J., Ward E., Forman D. (2011). Global cancer statistics. CA Cancer J. Clin.

[2] Hanahan D., Weinberg R.A. (2011). Hallmarks of cancer: the next generation. Cell.

[3] Bergers G., Benjamin L.E. (2003). Tumorigenesis and the angiogenic switch. Nat. Rev.

[4] Jain R.K. (1988). Determinants of tumor blood flow: A review. Cancer Res.

[5] Jain R.K., Munn L.L., Fukumura D. (2002). Dissecting tumour pathophysiology using intravital microscopy. Nat. Rev.

[6] Anderson S.A., Glod J., Arbab A.S., Noel M., Ashari P., Fine H.A., Frank J.A. (2005). Noninvasive MR imaging of magnetically labeled stem cells to directly identify neovasculature in a glioma model. Blood.

[7] Djonov V., Andres A.-C., Ziemiecki A. (2001). Vascular remodelling during the normal and malignant life cycle of the mammary gland. Microsc. Res. Tech.

[8] Brem S.S., Gullino P.M., Medina D. (1977). Angiogenesis: A marker for neoplastic transformation of mammary papillary hyperplasia. Science.

[9] Jensen H.M., Chen I., DeVault M.R., Lewis A.E. (1982). Angiogenesis induced by “normal” human breast tissue: a probable marker for precancer. Science.

[10] Guinebretiere J.M., le Monique G., Gavoille A., Bahi J., Contesso G. (1994). Angiogenesis and risk of breast cancer in women with fibrocystic disease. J. Natl. Cancer Inst.

[11] Weidner N., Folkman J., Pozza F., Bevilacqua P., Allred E.N., Moore D.H., Meli S., Gasparini G. (1992). Tumor angiogenesis: A new significant and independent prognostic indicator in early-stage breast carcinoma. J. Natl. Cancer Inst.

[12] Weidner N., Semple J.P., Welch W.R., Folkman J. (1991). Tumor angiogenesis and metastasis—correlation in invasive breast carcinoma. N. Engl. J. Med.

[13] Uzzan B., Nicolas P., Cucherat M., Perret G.Y. (2004). Microvessel density as a prognostic factor in women with breast cancer: a systematic review of the literature and meta-analysis. Cancer Res.

[14] Vincenti V., Cassano C., Rocchi M., Persico G. (1996). Assignment of the vascular endothelial growth factor gene to human chromosome 6p21.3. Circulation.

[15] Houck K.A. (1991). The vascular endothelial growth factor family: Identification of a fourth molecular species and characterization of alternative splicing of RNA. Mol. Endocrinol.

[16] Tischer E. (1991). The human gene for vascular endothelial growth factor. Multiple protein forms are encoded through alternative exon splicing. J. Biol. Chem.

[17] Neufeld G., Cohen T., Gengrinovitch S., Poltorak Z. (1999). Vascular endothelial growth factor (VEGF) and its receptors. FASEB J.

[18] Leung D.W., Cachianes G., Kuang W.J., Goeddel D.V., Ferrara N. (1989). Vascular endothelial growth factor is a secreted angiogenic mitogen. Science.

[19] Plouet J., Schilling J., Gospodarowicz D. (1989). Isolation and characterization of a newly identified endothelial cell mitogen produced by AtT20 cells. EMBO J.

[20] Nagy J.A. (2002). Vascular permeability factor/vascular endothelial growth factor induces lymphangiogenesis as well as angiogenesis. J. Exp. Med.

[21] Matsumoto T., Claesson-Welsh L. (2001). VEGF receptor signal transduction. Science STKE.

[22] Gerber H.P. (1998). VEGF regulates endothelial cell survival by the PI3-kinase/Akt signal transduction pathway. Requirement for Flk-1/KDR activation. J. Biol. Chem.

[23] Gerber H.P., Dixit V., Ferrara N. (1998). Vascular endothelial growth factor induces expression of the antiapoptotic proteins Bcl-2 and A1 in vascular endothelial cells. J. Biol. Chem.

[24] Gerber H.P. (1999). VEGF is required for growth and survival in neonatal mice. Development.

[25] Benjamin L.E., Golijanin D., Itin A., Pode D., Keshet E. (1999). Selective ablation of immature blood vessels in established human tumors follows vascular endothelial growth factor withdrawal. J. Clin. Invest.

[26] Yuan F. (1996). Time-dependent vascular regression and permeability changes in established human tumor xenografts induced by an anti-vascular endothelial growth factor/vascular permeability factor antibody. Proc. Natl. Acad. Sci. USA.

[27] Senger D.R. (1983). Tumor cells secrete a vascular permeability factor that promotes accumulation of ascites fluid. Science.

[28] Dvorak H.F., Brown L.F., Detmar M., Dvorak A.M. (1995). Vascular permeability factor/vascular endothelial growth factor, microvascular hyperpermeability, and angiogenesis. Am. J. Pathol.

[29] Bates D.O., Curry F.E. (1997). Vascular endothelial growth factor increases microvascular permeability via a Ca(2+)-dependent pathway. Am. J. Physiol.

[30] Roberts W.G., Palade G.E. (1995). Increased microvascular permeability and endothelial fenestration induced by vascular endothelial growth factor. J. Cell. Sci.

[31] Ku D.D., Zaleski J.K., Liu S., Brock T.A. (1993). Vascular endothelial growth factor induces EDRF-dependent relaxation in coronary arteries. Am. J. Physiol.

[32] Yang R. (1996). Effects of vascular endothelial growth factor on hemodynamics and cardiac performance. J. Cardiovasc. Pharmacol.

[33] Kim K.J. (1993). Inhibition of vascular endothelial growth factor-induced angiogenesis suppresses tumor growth *in vivo*. Nature.

[34] Ferrara N., Davis-Smyth T. (1997). The biology of vascular endothelial growth factor. Endocr. Rev.

[35] Fukumura D. (1998). Tumor induction of VEGF promoter activity in stromal cells. Cell.

[36] Gerber H.P., Kowalski J., Sherman D., Eberhard D.A., Ferrara N. (2000). Complete inhibition of rhabdomyosarcoma xenograft growth and neovascularization requires blockade of both tumor and host vascular endothelial growth factor. Cancer Res.

[37] Tsuzuki Y. (2000). Vascular endothelial growth factor (VEGF) modulation by targeting hypoxia-inducible factor-1[alpha→hypoxia response element→VEGF cascade differ entially regulates vascular response and growth rate in tumors. Cancer Res.

[38] Bergers G. (2000). Matrix metalloproteinase-9 triggers the angiogenic switch during carcinogenesis. Nat. Cell. Biol.

[39] Gasparini G. (2000). Prognostic value of vascular endothelial growth factor in breast cancer. Oncologist.

[40] Salven P., Perhoniemi V., Tykka H., Maenpaa H., Joensuu H. (1999). Serum VEGF levels in women with a benign breast tumor or breast cancer. Breast Cancer Res. Treat.

[41] Obermair A., Kucera E., Mayerhofer K., Speiser P., Seifert M., Czerwenka K., Kaider A., Leodolter S., Kainz C., Zeillinger R. (1997). Vascular endothelial growth factor (VEGF) in human breast cancer: correlation with disease-free survival. Int. J. Cancer.

[42] Ghosh S., Sullivan C.A., Zerkowski M.P., Molinaro A.M., Rimm D.L., Camp R.L., Chung G.G. (2008). High levels of vascular endothelial growth factor and its receptors (VEGFR-1, VEGFR-2, neuropilin-1) are associated with worse outcome in breast cancer. Human Pathol.

[43] Xie X.D., Qu S.X., Liu Z.Z., Zhang F., Zheng Z.D. (2009). Study on relationship between angiogenesis and micrometastases of peripheral blood in breast cancer. J. Cancer Res. Clin.

[44] Manders P., Beex L.V., Tjan-Heijnen V.C., Geurts-Moespot J., van Tienoven T.H., Foekens J.A., Sweep C.G. (2002). The prognostic value of vascular endothelial growth factor in 574 node-negative breast cancer patients who did not receive adjuvant systemic therapy. Br. J. Cancer.

[45] Bando H., Weich H.A., Brokelmann M., Horiguchi S., Funata N., Ogawa T., Toi M. (2005). Association between intratumoral free and total VEGF, soluble VEGFR-1, VEGFR-2 and prognosis in breast cancer. Br. J. Cancer.

[46] Toi M., Bando H., Ogawa T., Muta M., Hornig C., Weich H.A. (2002). Significance of vascular endothelial growth factor (VEGF)/soluble VEGF receptor-1 relationship in breast cancer. Int. J. Cancer.

[47] Ryden L., Stendahl M., Jonsson H., Emdin S., Bengtsson N.O., Landberg G. (2005). Tumor-specific VEGF-A and VEGFR2 in postmenopausal breast cancer patients with long-term follow-up. Implication of a link between VEGF pathway and tamoxifen response. Breast Cancer Res. Treat.

[48] Burstein H.J., Chen Y.H., Parker L.M., Savoie J., Younger J., Kuter I., Ryan P.D., Garber J.E., Chen H., Campos S.M. (2008). VEGF as a marker for outcome among advanced breast cancer patients receiving anti-VEGF therapy with bevacizumab and vinorelbine chemotherapy. Clin. Cancer Res.

[49] Brogan I.J., Khan N., Isaac K., Hutchinson J.A., Pravica V., Hutchinson I.V. (1999). Novel polymorphisms in the promoter and 5′ UTR regions of the human vascular endothelial growth factor gene. Hum. Immunol.

[50] Watson C.J., Webb N.J., Bottomley M.J., Brenchley P.E. (2000). Identification of polymorphisms within the vascular endothelial growth factor (VEGF) gene: Correlation with variation in VEGF protein production. Cytokine.

[51] Jin Q., Hemminki K., Enquist K., Lenner P., Grzybowska E., Klaes R., Henriksson R., Chen B., Pamula J., Pekala W. (2005). Vascular endothelial growth factor polymorphisms in relation to breast cancer development and prognosis. Clin. Cancer Res.

[52] Awata T., Inoue K., Kurihara S., Ohkubo T., Watanabe M., Inukai K., Inoue I., Katayama S. (2002). A common polymorphism in the 5′-untranslated region of the VEGF gene is associated with diabetic retinopathy in type 2 diabetes. Diabetes.

[53] Shahbazi M., Fryer A.A., Pravica V., Brogan I.J., Ramsay H.M., Hutchinson I.V., Harden P.N. (2002). Vascular endothelial growth factor gene polymorphisms are associated with acute renal allograft rejection. J. Am. Soc. Nephrol.

[54] Koukourakis M.I., Papazoglou D., Giatromanolaki A., Bougioukas G., Maltezos E., Sivridis E. (2004). VEGF gene sequence variation defines VEGF gene expression status and angiogenic activity in non-small cell lung cancer. Lung Cancer.

[55] Stevens A., Soden J., Brenchley P.E., Ralph S., Ray D.W. (2003). Haplotype analysis of the polymorphic human vascular endothelial growth factor gene promoter. Cancer Res.

[56] Pages G., Pouyssegur J. (2005). Transcriptional regulation of the Vascular Endothelial Growth Factor gene—a concert of activating factors. Cardiovasc. Res.

[57] Morris J.F., Hromas R., Rauscher F.J. (1994). Characterization of the DNA-binding properties of the myeloid zinc finger protein MZF1: two independent DNA-binding domains recognize two DNA consensus sequences with a common G-rich core. Mol. Cell. Biol..

[58] Awata T., Kurihara S., Takata N., Neda T., Iizuka H., Ohkubo T., Osaki M., Watanabe M., Nakashima Y., Inukai K. (2005). Functional VEGF C-634G polymorphism is associated with development of diabetic macular edema and correlated with macular retinal thickness in type 2 diabetes. Biochem. Biophys. Res. Commun.

[59] Huez I., Bornes S., Bresson D., Creancier L., Prats H. (2001). New vascular endothelial growth factor isoform generated by internal ribosome entry site-driven CUG translation initiation. Mol. Endocrinol.

[60] Krippl P., Langsenlehner U., Renner W., Yazdani-Biuki B., Wolf G., Wascher T.C., Paulweber B., Haas J., Samonigg H. (2003). A common 936 C/T gene polymorphism of vascular endothelial growth factor is associated with decreased breast cancer risk. Int. J. Cancer.

[61] Renner W., Kotschan S., Hoffmann C., Obermayer-Pietsch B., Pilger E. (2000). A common 936 C/T mutation in the gene for vascular endothelial growth factor is associated with vascular endothelial growth factor plasma levels. J. Vasc. Res.

[62] Doi K., Noiri E., Nakao A., Fujita T., Kobayashi S., Tokunaga K. (2006). Functional polymorphisms in the vascular endothelial growth factor gene are associated with development of end-stage renal disease in males. J. Am. Soc. Nephrol.

[63] Abe A., Sato K., Habuchi T., Wang L., Li Z., Tsuchiya N., Ohyama C., Satoh S., Ogawa O., Kato T. (2002). Single nucleotide polymorphisms in the 3′ untranslated region of vascular endothelial growth factor gene in Japanese population with or without renal cell carcinoma. Tohoku J. Exp. Med.

[64] Garcia-Closas M., Malats N., Real F.X., Yeager M., Welch R., Silverman D., Kogevinas M., Dosemeci M., Figueroa J., Chatterjee N. (2007). Large-scale evaluation of candidate genes identifies associations between VEGF polymorphisms and bladder cancer risk. PLoS Genet.

[65] Jacobs E.J., Feigelson H.S., Bain E.B., Brady K.A., Rodriguez C., Stevens V.L., Patel A.V., Thun M.J., Calle E.E. (2006). Polymorphisms in the vascular endothelial growth factor gene and breast cancer in the Cancer Prevention Study II cohort. Breast Cancer Res.

[66] Schneider B.P., Radovich M., Sledge G.W., Robarge J.D., Li L., Storniolo A.M., Lemler S., Nguyen A.T., Hancock B.A., Stout M. (2008). Association of polymorphisms of angiogenesis genes with breast cancer. Breast Cancer Res. Treat.

[67] Kidd L.R., Brock G.N., VanCleave T.T., Benford M.L., Lavender N.A., Kruer T.L., Wittliff J.L. (2010). Angiogenesis-associated sequence variants relative to breast cancer recurrence and survival. Cancer Causes Control.

[68] Langsenlehner U., Wolf G., Langsenlehner T., Gerger A., Hofmann G., Clar H., Wascher T.C., Paulweber B., Samonigg H., Krippl P. (2008). Genetic polymorphisms in the vascular endothelial growth factor gene and breast cancer risk. The Austrian “tumor of breast tissue: incidence, genetics, and environmental risk factors” study. Breast Cancer Res. Treat.

[69] Balasubramanian S.P., Cox A., Cross S.S., Higham S.E., Brown N.J., Reed M.W. (2007). Influence of VEGF-A gene variation and protein levels in breast cancer susceptibility and severity. Int. J. Cancer.

[70] Kataoka N., Cai Q., Wen W., Shu X.O., Jin F., Gao Y.T., Zheng W. (2006). Population-based case-control study of VEGF gene polymorphisms and breast cancer risk among Chinese women. Cancer Epidemiol. Biomark. Prev.

[71] Smith K.C., Bateman A.C., Fussell H.M., Howell W.M. (2004). Cytokine gene polymorphisms and breast cancer susceptibility and prognosis. Eur. J. Immunogenet.

[72] Oliveira C., Lourenco G.J., Silva P.M., Cardoso-Filho C., Favarelli M.H., Goncales N.S., Gurgel M.S., Lima C.S. (2011). Polymorphisms in the 5′- and 3′-untranslated region of the VEGF gene and sporadic breast cancer risk and clinicopathologic characteristics. Tumour Biol.

[73] Beeghly-Fadiel A., Shu X.O., Lu W., Long J., Cai Q., Xiang Y.B., Zheng Y., Zhao Z., Gu K., Gao Y.T. (2011). Genetic variation in VEGF family genes and breast cancer risk: A report from the Shanghai Breast Cancer Genetics Study. Cancer Epidemiol. Biomark. Prev.

[74] Eroglu A., Ozturk A., Cam R., Akar N. (2008). Vascular endothelial growth factor gene 936 C/T polymorphism in breast cancer patients. Med. Oncol.

[75] Gerger A., Langsenlehner U., Renner W., Weitzer W., Eder T., Yazdani-Biuki B., Hofmann G., Samonigg H., Krippl P. (2007). A multigenic approach to predict breast cancer risk. Breast Cancer Res. Treat.

[76] Jakubowska A., Gronwald J., Menkiszak J., Gorski B., Huzarski T., Byrski T., Edler L., Lubinski J., Scott R.J., Hamann U. (2008). The VEGF_936_C>T 3′UTR polymorphism reduces BRCA1-associated breast cancer risk in Polish women. Cancer Lett.

[77] Jakubowska A., Jaworska K., Cybulski C., Janicka A., Szymanska-Pasternak J., Lener M., Narod S.A., Lubinski J. (2009). Do BRCA1 modifiers also affect the risk of breast cancer in non-carriers?. Eur. J. Cancer.

[78] Rodrigues P., Furriol J., Tormo E., Ballester S., Lluch A., Eroles P. (2012). The single-nucleotide polymorphisms +936 C/T VEGF and −710 C/T VEGFR1 are associated with breast cancer protection in a Spanish population. Breast Cancer Res. Treat.

[79] Zhang B., Beeghly-Fadiel A., Lu W., Cai Q., Xiang Y.B., Zheng Y., Long J., Ye C., Gu K., Shu X.O. (2011). Evaluation of functional genetic variants for breast cancer risk: results from the Shanghai breast cancer study. Am. J. Epidemiol.

[80] Etienne-Grimaldi M.C., Formento P., Degeorges A., Pierga J.Y., Delva R., Pivot X., Dalenc F., Espie M., Veyret C., Formento J.L. (2011). Prospective analysis of the impact of VEGF-A gene polymorphisms on the pharmacodynamics of bevacizumab-based therapy in metastatic breast cancer patients. Br. J. Clin. Pharmacol.

[81] Lu H., Shu X.O., Cui Y., Kataoka N., Wen W., Cai Q., Ruan Z.X., Gao Y.T., Zheng W. (2005). Association of genetic polymorphisms in the VEGF gene with breast cancer survival. Cancer Res.

[82] Wolf G., Aigner R.M., Schaffler G., Langsenlehner U., Renner W., Samonigg H., Yazdani-Biuki B., Krippl P. (2004). The 936C>T polymorphism of the gene for vascular endothelial growth factor is associated with 18F-fluorodeoxyglucose uptake. Breast Cancer Res. Treat.

[83] Knechtel G., Hofmann G., Gerger A., Renner W., Langsenlehner T., Szkandera J., Wolf G., Samonigg H., Krippl P., Langsenlehner U. (2010). Analysis of common germline polymorphisms as prognostic factors in patients with lymph node-positive breast cancer. J. Cancer Res. Clin.

[84] Wang K., Liu L., Zhu Z.M., Shao J.H., Xin L. (2011). Five polymorphisms of vascular endothelial growth factor (VEGF) and risk of breast cancer: a meta-analysis involving 16,703 individuals. Cytokine.

[85] Yang D.S., Park K.H., Woo O.H., Woo S.U., Kim A.R., Lee E.S., Lee J.B., Kim Y.H., Kim J.S., Seo J.H. (2011). Association of a vascular endothelial growth factor gene 936 C/T polymorphism with breast cancer risk: a meta-analysis. Breast Cancer Res. Treat.

[86] Gu D., Wang M. (2011). VEGF 936C>T polymorphism and breast cancer risk: evidence from 5729 cases and 5868 controls. Breast Cancer Res. Treat.

[87] Liu L., Liu L., Zeng F., Wang K., Huang J., Xin L., Zhu P.Q. (2011). Meta-analysis of the association between VEGF-634 G>C and risk of malignancy based on 23 case-control studies. J. Cancer Res. Clin.

[88] Miller K., Wang M., Gralow J., Dickler M., Cobleigh M., Perez E.A., Shenkier T., Cella D., Davidson N.E. (2007). Paclitaxel plus bevacizumab *versus* paclitaxel alone for metastatic breast cancer. N. Engl. J. Med.

[89] Miles D.W., Chan A., Dirix L.Y., Cortes J., Pivot X., Tomczak P., Delozier T., Sohn J.H., Provencher L., Puglisi F. (2010). Phase III study of bevacizumab plus docetaxel compared with placebo plus docetaxel for the first-line treatment of human epidermal growth factor receptor 2-negative metastatic breast cancer. J. Clin. Oncol.

[90] Thomssen C., Pierga J.Y., Pritchard K.I., Biganzoli L., Cortes-Funes H., Petrakova K., Kaufman B., Duenne A., Smith I. (2012). First-line bevacizumab-containing therapy for triple-negative breast cancer: analysis of 585 patients treated in the ATHENA study. Oncology.

[91] Miller K.D., Chap L.I., Holmes F.A., Cobleigh M.A., Marcom P.K., Fehrenbacher L., Dickler M., Overmoyer B.A., Reimann J.D. (2005). Randomized phase III trial of capecitabine compared with bevacizumab plus capecitabine in patients with previously treated metastatic breast cancer. J. Clin. Oncol.

[92] Kostopoulos I., Arapantoni-Dadioti P., Gogas H., Papadopoulos S., Malamou-Mitsi V., Scopa C.D., Markaki S., Karagianni E., Kyriakou V., Margariti A. (2006). Evaluation of the prognostic value of HER-2 and VEGF in breast cancer patients participating in a randomized study with dose-dense sequential adjuvant chemotherapy. Breast Cancer Res. Treat.

[93] Ludovini V., Sidoni A., Pistola L., Bellezza G., de Angelis V., Gori S., Mosconi A.M., Bisagni G., Cherubini R., Bian A.R. (2003). Evaluation of the prognostic role of vascular endothelial growth factor and microvessel density in stages I and II breast cancer patients. Breast Cancer Res. Treat.

[94] MacConmara M., O’Hanlon D.M., Kiely M.J., Connolly Y., Jeffers M., Keane F.B. (2002). An evaluation of the prognostic significance of vascular endothelial growth factor in node positive primary breast carcinoma. Int. J. Oncol.

[95] Schneider B.P., Wang M., Radovich M., Sledge G.W., Badve S., Thor A., Flockhart D.A., Hancock B., Davidson N., Gralow J. (2008). Association of vascular endothelial growth factor and vascular endothelial growth factor receptor-2 genetic polymorphisms with outcome in a trial of paclitaxel compared with paclitaxel plus bevacizumab in advanced breast cancer: ECOG 2100. J. Clin. Oncol.

